# The Establishment of an Indigenous‐Led Drinking Water Monitoring Program Leveraging qPCR and Metagenomics Testing in New Zealand

**DOI:** 10.1002/wer.70471

**Published:** 2026-06-27

**Authors:** Connor Redmile, Donna Sutherland, Megan Devane, William Taylor, Isobel Busby, Anne Glackin, Brent Gilpin, Tim Chambers

**Affiliations:** ^1^ Te Kura Taka Pini Ltd. Christchurch New Zealand; ^2^ New Zealand Institute for Public Health and Forensic Science New Zealand; ^3^ Ngāi Tahu Research Centre University of Canterbury, Te Whare Wānanga O Waitaha Christchurch, Ōtautahi New Zealand

**Keywords:** drinking water, freshwater, indigenous‐led, metagenomics, public health

## Abstract

An Indigenous‐led monitoring program was established in partnership with the South Island Māori (Indigenous population of New Zealand [NZ]) tribe of NZ to understand and improve local drinking water safety. The aims of the project were to: (1) establish an Indigenous‐led drinking water monitoring program; (2) utilize a full suite of monitoring tools to understand source water hazards and treatment efficacy; and (3) test the effectiveness of advanced water sampling techniques in Indigenous communities. Advanced sampling techniques were used for fecal source tracking to identify existing public health hazards and to provide assurance that any remedial interventions were effective. The program trained a total of 27 individuals from 16 different Indigenous communities in water quality sampling and helped to identify and address six microbial water quality issues. This project underscored the benefits of engaging Indigenous Peoples in governance and decision‐making processes and in alleviating systemic barriers that prevent Indigenous communities from realizing safe water quality and sufficient water infrastructure.

## Summary

- Investing in the capability and capacity of local Indigenous communities can facilitate large‐scale water quality monitoring programs.
- Indigenous‐led approaches can effectively direct drinking water infrastructure upgrades.
- Advanced sampling techniques can be integrated in a rigorous community‐based testing program with sufficient specialist support and guidance.
- The use of qPCR and metagenomic testing can help to identify and address sources of microbial contamination.
- Metagenomic testing is still developing; however, this monitoring tool shows great promise for applications in the water sector.

## Graphical abstract

An Indigenous‐led monitoring program was established in the South Island of New Zealand to improve local drinking water safety. Advanced sampling techniques were used to identify public health risks and provide assurance that remedial interventions were effective. Individuals from Indigenous communities were upskilled, and significant microbial issues were resolved.

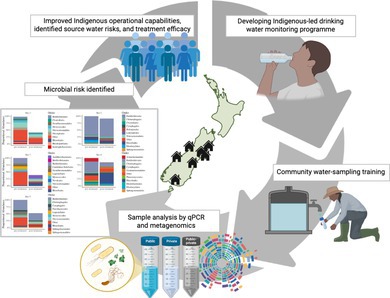

## 1 Introduction

Indigenous Peoples have long relied on freshwater to shape identity, to support livelihoods, and to sustain social and cultural prosperity. Indigenous Peoples are reliant on the aquatic environment for sustenance and are particularly vulnerable to freshwater degradation and the associated illnesses from ingesting contaminants (Balasooriya et al. 2023). Freshwater environments in Indigenous spaces are increasingly degraded due to wastewater discharge, stormwater runoff, and agricultural pollution, causing elevated levels of chemical pollutants (e.g., nitrates and heavy metals), microbial pollution from fecal sources (e.g., *Escherichia coli* , *Salmonella*, and *Campylobacter*), emerging contaminants, and sediments (Chakravarthy et al. 2019; Rogers et al. 2025; Simmons et al. 2001).

Fecal source tracking tools are used to identify sources of fecal contamination in water bodies. These tools include microbial source tracking (MST) markers, which use genetic methods to identify microorganisms or DNA of the host animal (Demeter et al. 2023; Cookson et al. 2024). Microbial markers target fecal microbes, which are highly associated with a particular animal species (but not always unique to) that animal host (Ahmed et al. 2019; Reischer et al. 2006). In comparison to MST markers, metagenomics is a relatively new tool with the potential to target the entire microbial population in drinking water, including bacteria, viruses, and protozoa (Brumfield et al. 2020; Mahajna et al. 2022; Putri et al. 2024). Metagenomics will potentially enable better detection of pathogens and identify changes in drinking water communities that represent real health hazards.

Indigenous Peoples are disproportionately affected by water quality decline (Mattos et al. 2021), in which the health impacts documented by Indigenous Peoples mainly derive from polluted water and food (Bradford et al. 2016; Dudarev et al. 2013; Huseman and Short 2012; King et al. 2013). They have managed and governed natural systems since time immemorial through relationships founded on values of respect and reciprocity (Wilson et al. 2018). Many Indigenous Peoples view water as a living being or ancestor for whom they have a responsibility to protect (Acharibasam et al. 2024). Zúñiga (2025) has illustrated the significance of a worldview by stating that, “The manner in which living beings (human and more‐than‐human) represent and think the world informs how they act in it and why—in other words, it informs their ethical practice”. The contemporary governance frameworks that have been imposed on Indigenous Peoples over natural resources are therefore biased against them, due to philosophical differences about human‐nature relationships, different methods of environmental knowledge generation, misaligned use patterns and priorities, and desires for tribal sovereignty (Gadamus and Raymond‐Yakoubian 2015; Rout et al. 2017).

For Māori, the Indigenous People of New Zealand (NZ), water is part of whakapapa: a larger, complex system of genealogical relationships encompassing more than just biological objects (environments and sentient beings), including material, spiritual, and historical elements that are interconnected across space and time (Stewart‐Harawira 2020). Water has always been central to Ngāi Tahu (the tribal group of the South Island, NZ) way of life; travel routes were dictated by rivers and streams, spring waters were reserved for cleansing, specific rivers were designated for burial sites (Bonis et al. 2006), and mahinga kai (representing traditional foods, their sources, and methods of gathering) practices were centered around significant rivers, lakes, and wetlands (Timms‐Dean et al. 2024). For example, Te Waihora, previously known as “The Fish Basket of Rākaihautū,” is a lake central to Ngāi Tahu mahinga kai. As indicated by the name, the lake is revered for the historical abundance of fish, shellfish, birds, flaxes, sedges, seeds, and trees, all used for various purposes including housing and canoe construction, weaving, dyeing fiber, food, medicine, tools, and personal ornaments (Te Waihora Co‐Governance 2025). The health and prosperity of Ngāi Tahu communities are therefore interdependent with the health of waterbodies.

Māori rights and interests remain limited in NZ, a challenge borne by Indigenous populations globally. This imbalance is primarily due to an inadequate recognition of Māori authority in resource management. Land use in NZ is predominantly regulated by regional councils (local government) that operate under a framework set by the central government; rules are prescribed in the Resource Management Act 1991 (RMA) allowing for regional councils to use, take, dam, and discharge contaminants into water (Alex 2018). While the Treaty of Waitangi (1840) affirms Māori rangatiratanga (authority) over taonga (treasure) such as freshwater, legislation does not provide substantive Māori governance or decision‐making powers. Instead, Māori input into decision‐making processes is generally still limited to non‐binding advisory or consultation processes (Tipa 2003). The inadequacy of these processes is currently the subject of a high court case between Ngāi Tahu and the NZ Government. Ngāi Tahu are seeking legal recognition of their rangatiratanga over freshwater within their tribal area and declarations that the Crown ought to work in partnership with Ngāi Tahu to design a better system to manage the freshwater degradation that has resulted from decades of poor resource management (Tau v Attorney‐General CIV‐2020‐409‐534 [pending judgment], n.d.).

For Ngāi Tahu, marae commonly encompass a complex of buildings for tangata whenua (people of the land) to connect with tūpuna (ancestors), whānau (family; community of families), and manuhiri (guests), usually founded on Māori land reserves (Bennett 2007). They are unique social infrastructure in NZ that foster and maintain social networks, tradition, and tribal economies. As such, marae experience fluctuations in attendance for celebrations, ceremonies, and gatherings. Access to quality and reliable drinking water infrastructure is central to ensuring these marae fulfill important sociocultural roles while maintaining public health priorities.

Ngāi Tahu communities connected to public supplies must assume that the safety of water services is adequate because their authority and inclusion in decision‐making remain limited. However, simply meeting the drinking water standards is often misaligned with community aspirations and does not always provide adequate protection. For example, a rural Māori community has been required to utilize unsatisfactory drinking water from their local municipality supply, which breached the *Water Services (Drinking Water Standards for New Zealand) Regulations* 2022 *(SL 2022/168)* 2022 for nitrate and had to be temporarily disconnected in 2022 (Prickett et al. 2024). Further municipality failures across NZ have occurred in Havelock North (Graham et al. 2023; McLaren et al. 2022), Waikouaiti (Uwins‐England and McKenzie 2021), and Queenstown (Baker et al. 2023), often with severe health consequences.

Indigenous communities globally have spearheaded water quality monitoring programs to overcome the systemic constraints that inhibit their engagement in the governance of freshwater systems and to protect their lands (Wilson et al. 2018). It is crucial that Indigenous Peoples' worldviews and leadership positions are promoted to enhance their access to safe drinking water (Acharibasam et al. 2024). Wilson et al. (2018) previously highlighted that Indigenous Peoples can use community‐collected water data to assert self‐determination in decision‐making processes. Deliberate opportunities for Indigenous leadership and data governance are therefore fundamental in ensuring that Indigenous Peoples are not exposed to extractive approaches to knowledge generation and sharing. Empowering Indigenous Peoples to govern the exchange and integration of knowledge and data will encourage a more holistic understanding of water quality and improve trust relationships (Wilson 2025; Wilson et al. 2018).

Public distrust in governments or industry often gives rise to “bottom‐up” community projects. Indigenous bottom‐up approaches are advantageous for addressing local concerns and holding governance to account. For example, 12 Indigenous‐led monitoring projects of the Mackenzie River, Canada, used a range of different monitoring methods and tools to address their own local concerns, such as the immediate safety of eating and drinking from the river in their area (Parlee et al. 2021). Furthermore, Parlee et al. (2021) and Wilson et al. (2018) have highlighted that a bottom‐up approach addresses accountability of governance—a concept often overlooked by colonial governments—by positioning the approach as one that values and is responsive to local community concerns.

Several monitoring programs have emphasized the benefits of capacity building and increasing accessibility in Indigenous‐led water quality monitoring. These benefits were mostly achieved by providing equipment, technical training, and knowledge sharing opportunities for skill development as well as enabling long‐term program viability (Herman‐Mercer et al. 2018; O'Connor et al. 2022). For example, the Manitoba Métis Federation employed a self‐governed approach to Red River monitoring, allowing it to be carried out through a citizen science initiative (Combe 2023). This program design has provided opportunities to strengthen environmental stewardship, support intergenerational knowledge transfers, and reinforce Indigenous sovereignty over environmental decision‐making (Acharibasam et al. 2024; Baijus 2021).

Many studies have highlighted that while water monitoring projects were successful, they were often threatened by the long‐term economic feasibility of operations. For instance, the remote Arctic environment of Tuktoyaktuk exemplified that the high financial costs of travel, fieldwork, and shipping, combined with the lack of infrastructure and short‐term funding structures, were challenges to long‐term water quality monitoring efforts (Mercer et al. 2025). In addition, bottom‐up approaches can lead to governments expressing quality and credibility concerns (Buckland‐Nicks et al. 2016; Hunsberger 2004). However, the demand for Indigenous‐led solutions is only expected to increase, so it is imperative that affordable and accessible solutions are explored.

The aims of this research were to: (1) establish an Indigenous‐led drinking water monitoring program; (2) utilize a full suite of monitoring tools to understand source water hazards and treatment efficacy; and (3) test the effectiveness of advanced water sampling techniques in Indigenous communities. These objectives were designed to understand and improve the quality of drinking water consumed at Ngāi Tahu marae and to strengthen local capabilities that sustained improvements made in water safety.

## 2 Methods

### 2.1 Study Locations

**FIGURE 1:**
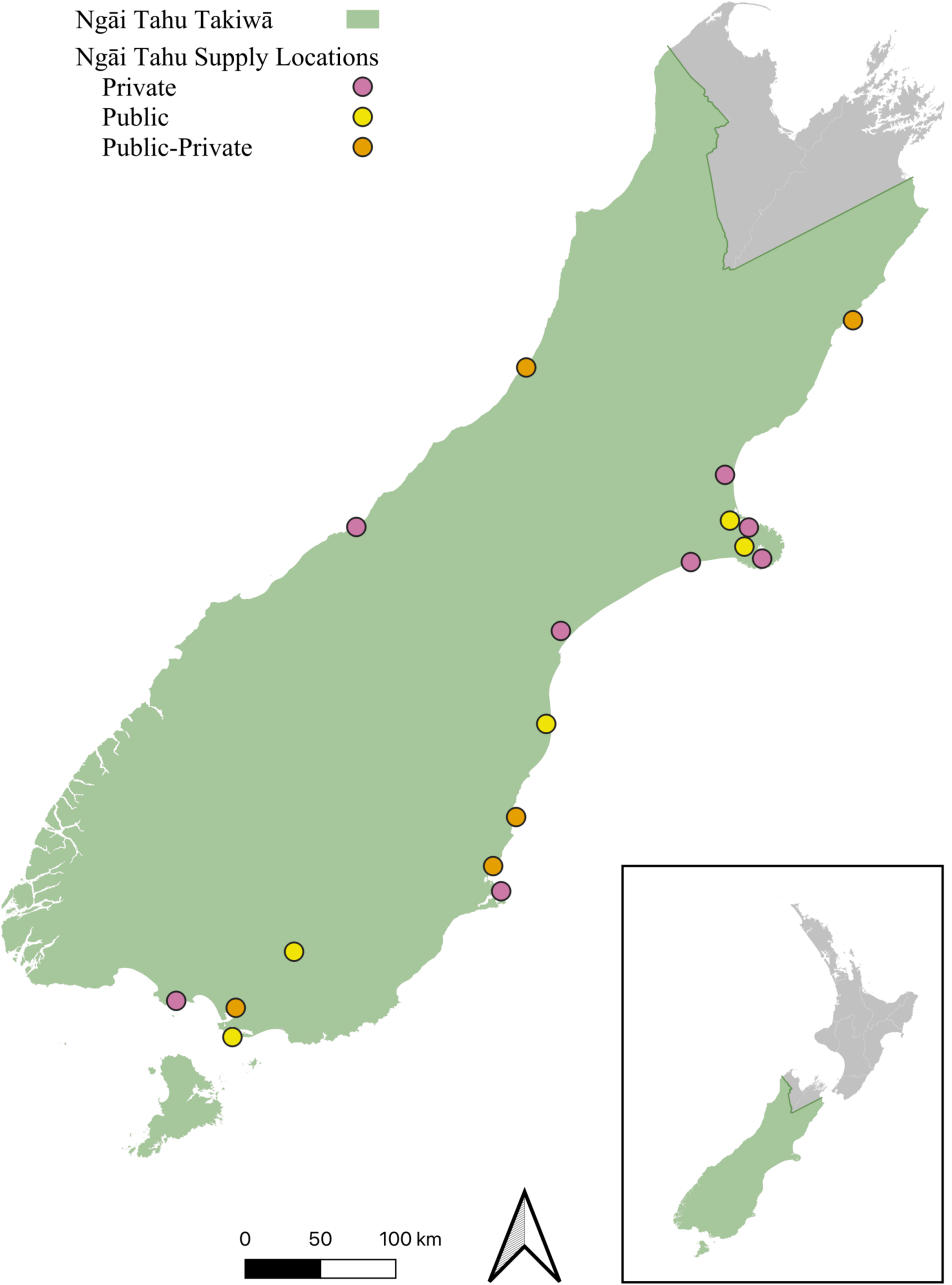
Map of Ngāi Tahu Takiwā and Location of Sites. *Note:* The takiwā of Ngāi Tahu is defined in the Te Runanga o Ngai Tahu Act 1996.

This study included the Ngāi Tahu takiwā (tribal boundary) that covers the majority of the South Island of NZ (Figure 1). The 17 marae and one papakāinga (a community of at least three dwellings on Māori land) included in this study are primarily located along the coast, with very few inland.

### 2.2 Classification of Drinking Water Systems

To facilitate analysis, broad categories of the diverse drinking water systems used at marae and the papakāinga were established. These categories ensured anonymity of data analysis and upheld data sovereignty principles. The three categories of ownership type were defined as (a) public: water supply is provided entirely through a water supply registered with Taumata Arowai|The Water Services Authority; (b) public‐private: water is received from a public supply but is stored on site and/or is supplemented by an additional collection method; and (c) private: drinking water is collected, stored, and treated with no input from public water suppliers. The source of each drinking water supply was classified as either groundwater, surface water, or rainwater. When a supply had multiple sources, the primary source based on quantity proportions was used.

### 2.3 Monitoring Program

A systematic and comprehensive Ngāi Tahu Monitoring Program (NTMP) was developed to investigate drinking water quality, efficacy of treatment, and capability and capacity at 17 Ngāi Tahu marae and one papakāinga. The program was led by Te Kura Taka Pini (the specialist water entity of Ngāi Tahu) with support from the University of Canterbury and the New Zealand Institute for Public Health and Forensic Science. The program was an opportunity to evaluate microbial contaminants in community drinking water supplies across the Ngāi Tahu takiwā, in line with local aspirations seeking to provide safe drinking water. The sites were selected for monitoring due to their cultural importance as well as centrality in Māori communities, serving as sentinel sites of community drinking water quality.

### 2.4 Recruitment of Māngai Wai Māori

The NTMP required appropriate operational support to facilitate sampling. Local water champions were recruited to support the monitoring program across a large geographical region. A Ngāi Tahu upoko (chief) gifted the name Māngai Wai Māori (MWM) to this role, which translates to a representative of freshwater. This title better aligned with Māori worldviews and made the role more significant to the recruited representatives. The MWM role was established so that it could evolve in the future and integrate the broader aspirations of each local community and the needs of the tribe. Each of the MWM was compensated $3750 (NZD) on average to cover training time, participation in four sampling sessions, additional onsite support/visits, and average mileage/travel reimbursements. This approach ensured their time and effort were valued appropriately and that costs did not present a prohibitive barrier.

### 2.5 Training for MWM

#### 2.5.1 Bespoke Online Course

The development of a training program was completed in collaboration with Water New Zealand, a water industry representative group, using their existing online training infrastructure through the Articulate 360 Platform. A bespoke course was configured by repurposing existing content that related to drinking water monitoring and freshwater science from Water New Zealand courses. Additional training content tailored for the Ngāi Tahu context was generated with in‐house expertise. Two short videos were curated to encourage engagement with the taught content and to overcome educational barriers. This included a bespoke module with a video on “The Importance of Wai Māori (freshwater) to Ngāi Tahu” and a second video that demonstrated a step‐by‐step guide on “How to Take a Routine Water Sample,” which could be replayed when sampling. The course material has not been made publicly available to safeguard the commercial interests of Water New Zealand, as some of the module content has been repurposed from their existing paid courses.

#### 2.5.2 Protocol Provision

A bespoke sampling protocol consolidated established methods and practices, including the Hill Labs and Christchurch City Council protocols (see Supporting Information). The protocol included detailed instructions to collect water samples with a 500 mL unpreserved container, 400 mL sterile container, and 100 mL nitric acid preserved container required for the routine water test. The protocol was also used to inform the script for the complementary sampling video. This ensured that MWM could visibly see what was required by each step of the protocol to ensure repeatability of a consistent method across the takiwā and remove subjective interpretations of nuanced instructions.

#### 2.5.3 One‐on‐One Demonstration: Site Visits

Each of the 18 sites was visited by one or two researchers with the purpose of meeting MWM face‐to‐face during the first round of drinking water sampling. MWM were encouraged to independently complete the sampling under the researchers' supervision and to ask for help when they required additional support. The prescribed sampling protocol was followed at each site. After water samples were completed, they were delivered directly or via courier services to an International Accreditation New Zealand accredited laboratory for relevant analysis.

### 2.6 Sampling Program Overview

Four rounds of drinking water sampling for fecal indicator bacteria were completed over 12 months. During the first water quality sample, an additional routine drinking water sample post‐treatment was completed to characterize the water chemistry and identify any non‐microbial contaminants. The second sampling event, completed 3 months after the first, utilized quantitative polymerase chain reaction (qPCR) for fecal source tracking and metagenomic testing for a proof‐of‐concept study to evaluate the efficacy of using novel genetic tools to supplement information from regulatory testing for fecal indicator bacteria. For the supplies that were operating a private drinking water supply, a pre‐ and post‐treatment sample was taken where access allowed. Below is an outline of the different testing methods utilized for the different components of the program.

#### 2.6.1 Routine Drinking Water Sample

Conducted by Hill Labs for fecal indicator bacterial and chemical contaminants required for compliance with the *Water Services (Drinking Water Standards for New Zealand) Regulations* 2022 *(SL 2022/168)* 2022. See Table 1 for specific information on the tests and detection limits. Each sample was required to be delivered to the nearest testing laboratory within 24 h of sampling and between 4°C–10°C. The samples were held at Hill Labs for a length of time based on the stability of samples, analytes being tested, and the storage space available. Hill Labs processed the samples in accordance with the terms of their accreditation.

**TABLE 1:** Routine water testing methods and detection limits.

Determinand	Method description	Detection limit	NZ MAV
*Escherichia coli*	MPN count using Colilert 18 (IDEXX, Laboratories Inc., Maine, US) (Incubated at 35°C for 24 h) and 97 wells. Analyzed at Hill Laboratories—Microbiology; Unit 1, 17 Print Place, Middleton, Christchurch. APHA 9223 B: Online Edition.	1 MPN/100 mL	< 1 MPN/100 mL
Total arsenic	Nitric acid digestion, ICP‐MS, trace level. APHA 3125 B: Online 1 Edition/US EPA 200.8.	0.0011 g/m3	0.01 g/m3
Total boron	Nitric acid digestion, ICP‐MS, trace level. APHA 3125 B: Online 1 Edition.	0.0053 g/m3	2.4 g/m3
Total copper	Nitric acid digestion, ICP‐MS, trace level. APHA 3125 B: Online 1 Edition/US EPA 200.8.	0.00053 g/m3	2.0 g/m3
Total lead	Nitric acid digestion, ICP‐MS, trace level. APHA 3125 B: Online 1 Edition/US EPA 200.8.	0.00011 g/m3	0.01 g/m3
Nitrate‐N	Filtered (if required) sample from Christchurch. Ion Chromatography. APHA 4110 B (modified): Online Edition.	0.05 g/m3	11.3 g/m3
Total Manganese	Nitric acid digestion, ICP‐MS, trace level. APHA 3125 B: Online 1 Edition/US EPA 200.8.	0.00053 g/m3	0.4 g/m3

#### 2.6.2 Enumeration of Fecal Indicator Bacteria in Drinking Water

The detection and quantification of fecal indicator bacteria ( *Escherichia coli* and total coliforms) was undertaken by testing 100 mL of drinking water using the Colilert system for enzyme‐substrate coliform testing with a 97‐well Most Probable Number (MPN) Quantitray method (IDEXX, Maine, USA).

#### 2.6.3 MST Markers

The water samples were tested using MST markers to differentiate between fecal sources, displayed in Table 2. Untreated source water and un/treated drinking water samples (1 L–2 L) were filtered through 0.45 μm mixed cellulose ester membrane filters (Millipore, France) and DNA was extracted using the PowerSoil Pro kit (Qiagen, Venlo, The Netherlands) as described previously (Cookson et al. 2024). All sampling events included filter blanks, distilled water blanks, and DNA extraction reagent blanks. The qPCR analysis was performed on a LightCycler 480 (Roche Diagnostics Ltd., California, US), and the MST qPCR targets are presented in Table 2, with PCR protocols and conditions as described previously (Cookson et al. 2024). Each qPCR run included negative and positive controls, and standard curves generated from 10‐fold serial dilutions of the appropriate MST target with a range of quantification from 10^0^ to 10^6^ Gene Copies/100 mL. PCR assays were considered acceptable at > 90% amplification efficiency and with a coefficient of determination (*r* ^2^) at ≥ 0.92 for assays.

**TABLE 2:** Target microbial genes and methods for microbial source tracking using qPCR.

Fecal source host/target	Microbial target	Type of qPCR assay	References
General	Bacteroidales 16S rRNA (GenBac)	Probe‐based	(Siefring et al. 2008)
Human	Duplex qPCR of *Bacteroides* HF183 and crAssphage CPQ_056	Probe‐based	(Ahmed et al. 2019)
	*Bifidobacterium adolescentis* (BiADO)	SYBR Green	(Matsuki et al. 2004)
Ruminant^a^	Bacteroidales 16S rRNA (BacR)	Probe‐based	(Reischer et al. 2006)
Avian^b^	GFD – Unclassified *Helicobacter* 16S rRNA gene	SYBR Green	(Green et al. 2012)

^a^ Targets cattle, sheep, deer, and goats.

^b^ Targets wildfowl (swans, gulls, ducks, Canada Goose, and Pūkeko).

#### 2.6.4 Metagenomic Testing

DNA concentrations of drinking water extracts were measured with a Qubit 4.0 Fluorometer (Thermo Fisher Scientific, Waltham, Massachusetts, US). Primers (27FII: 5‐AGRGTTYGATYMTGGCTCAG‐3, 1492R: 5‐CGGYTACCTTGTTACGACTT‐3) were used to amplify the full‐length 16S rRNA gene (~1500 base pairs) used for bacterial identification (Matsuo et al. 2021; Waechter et al. 2023). The PCR products were input to libraries that were prepared using the Native Barcoding Kit (SQK‐NBD114.96, Oxford Nanopore Technologies, Oxford, United Kingdom) and PCR‐Expansion Barcoding Kit (EXP‐PCB096). Sequencing was performed on a GridION using R10.1.4 flow cells. Samples were demultiplexed with Dorado (version 1.1.0; Oxford Nanopore Technologies 2025), primers were removed with cutadapt (version 4.5; Martin 2011), and reads were filtered using Filtlong (version 0.2.1; Wick 2021), retaining only those between 1300 and 1600 base pairs with a mean q‐score above 80. Finally, samples underwent taxonomic assignment using Emu version 3.5.1 and the version 3.4.5 database to identify bacteria in the samples (Curry et al. 2022). All sequencing runs included the following blanks: extraction blanks for filters, reagents (no template controls, NTC), and DNA extraction and sequencing amplification. After sequencing, blanks were reviewed for contamination, and any potential contaminant reads were removed. Sequences were not uploaded to a public database, as the Māori communities retain ownership over the data. As such, the data is stored on a local server hosted in New Zealand and governed by Te Kura Taka Pini.

### 2.7 Remedial Interventions

Upon the identification of water quality issues following water sampling, internal technical expertise provided advice and supported the implementation of remedial interventions to improve drinking water quality. The metagenomic results are presented before and after the remedial intervention for one case study where microbial contamination was positive. Each intervention followed a process of investigation and resolution. Investigative sampling helped isolate the cause of each issue, such as improper maintenance or system design faults. The resolutions were tailored to address each issue, including: a deep clean of rooftop gutters and/or water storage tanks; the replacement of nonfunctional ultraviolet (UV) treatment systems or installation of new ones; recommendations to change operating procedures (e.g., maintenance program, flushing periods, and replacement schedule); and ongoing monitoring to test the interventions' effectiveness.

### 2.8 Analysis

Descriptive statistics for the drinking water quality from routine water testing were summarized by water supply type. The minimum and maximum values were presented for each water supply category, as well as the number of locations that were above the maximum acceptable value (MAV) and half of this value as specified by the *Water Services (Drinking Water Standards for New Zealand) Regulations* 2022 *(SL 2022/168)* 2022.

Metagenomic analysis was performed in R (version 4.3.3; R Core Team 2025) using the phyloseq package (version 1.50.0; McMurdie and Holmes 2013). Species were filtered for those represented by at least 50 reads in a sample. Scaling values for proportional abundance were calculated by dividing the number of species in pre‐treatment samples by the number of species in post‐treatment samples and multiplying the abundance values by the scaling value for each site. Source attribution was based on a curated assessment from the FAPROTAX database (Louca et al. 2016) using species designations from MicrobeAtlas (Matias Rodrigues et al. 2026) and an assessment of the published literature for habitat associations for bacterial species.

## 3 Results

### 3.1 Māngai Wai Māori and Locations

There were 27 MWM trained in total, representing 15 marae and one papakāinga. Members of the research team supported water sampling for two marae so that all 18 sub‐tribal areas were included (Table 3). The sites received water from either a public supply (*n* = 5), a public‐private supply (*n* = 5), or a private supply (*n* = 8). Groundwater was the most common source (*n* = 9), followed by surface water (*n* = 6) and then rainwater (*n* = 3).

**TABLE 3:** Water supply type and source water.

Supply type	Source water			
	Groundwater	Rainwater	Surface water	Total
Public	3	0	2	5
Public‐private	2	0	3	5
Private	4	3	1	8
Total	9	3	6	18

### 3.2 Routine Drinking Water Quality Results

Table 4 provides an overview of the routine drinking water quality testing results. For the public supplies, there were no exceedances of the MAV for *E. coli* but there was a detection for total coliforms. There were also no detections for any chemical drinking water indicators exceeding the MAV in public supplies, although one sample exceeded half the MAV for nitrate‐N (6.4 mg/L). Across both public‐private and private supplies, there were four locations with detections for *E. coli* and five locations for total coliforms. The maximum *E. coli* detection in public‐private supplies was 1 per 100/mL sample, while for total coliforms a maximum of 378 per 100/mL sample was measured. In private supplies, the highest detected concentration of *E. coli* and total coliforms was 18 and 980 per 100/mL, respectively. There were no detections for chemical drinking water indicators above half the MAV in public‐private or private supplies.

**TABLE 4:** Routine water testing results for Ngāi Tahu sampling locations.

Determinand	Public				Public‐private				Private			
	Min	Max	> MAV^a^	> ½ MAV	Min	Max	> MAV	> ½ MAV	Min	Max	> MAV	> ½ MAV
*E. coli*	0	0	0	0	0	1	2	2	0	18	2	2
Total coliforms^b^	0	5	1	1	0	378	2	2	0	980	3	3
Nitrate‐N	0.21	6.4	0	1	0	0.78	0	0	0.08	4.4	0	0
Total arsenic	0	0	0	0	0	0	0	0	0	0	0	0
Total boron	0.15	0.032	0	0	0	0.032	0	0	0	0.115	0	0
Total copper	0.006	0.024	0	0	0.003	0.076	0	0	0.001	0.33	0	0
Total lead	0	0.002	0	0	0	0.001	0	0	0	0.004	0	0
Total manganese	0	0.001	0	0	0	0.002	0	0	0	0.003	0	0

*Note:* Number of public supplies = 5, number of public‐private supplies = 5, number of private supplies = 8, total number = 18.

^a^ Number of locations that had a result that was above the MAV or guideline value.

^b^ Not technically a legislated MAV but there is a guideline value.

### 3.3 qPCR and Metagenomic Results

#### 3.3.1 Fecal Source Markers

Fecal‐associated bacteria and the general fecal source marker and/or animal‐specific markers were identified in the post‐treatment samples from several sites as well as *E. coli* and total coliforms (see Table S1). Of the post‐treatment samples, no human or avian markers were detected. In total, eight post treatment samples had general fecal maker detections; of these, three had *E. coli* and five had total coliform detections. In two post‐treatment samples, ruminant markers were detected, alongside the general fecal source marker and total coliform detections but not *E. coli* .

#### 3.3.2 Types of Environments Inhabited by Bacteria

**FIGURE 2:**
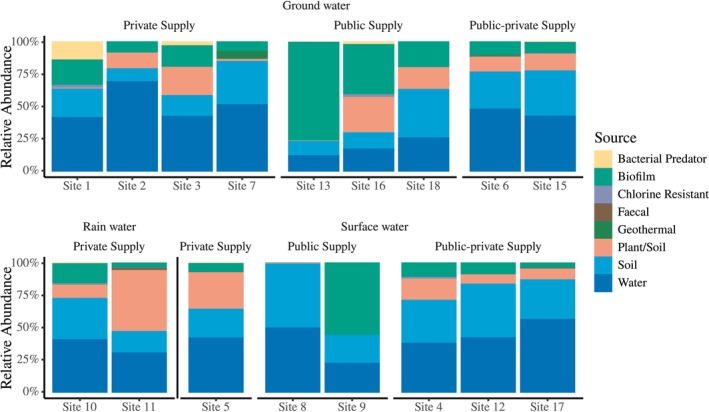
Type of environments associated with bacteria in post‐treated waters from each site.

The type of environments associated with bacteria or characteristic of bacteria detected in the post‐treatment water samples from the 18 sites is shown in Figure 2, arranged by supply type. Most of the bacterial environments were consistent with expected results, including those detected in groundwater. There was a high relative abundance of bacteria that were associated with biofilm environments detected in the public water supplies, which could indicate that established biofilm communities in the pipe systems were being detected.

#### 3.3.3 Treatment Effectiveness

**FIGURE 3:**
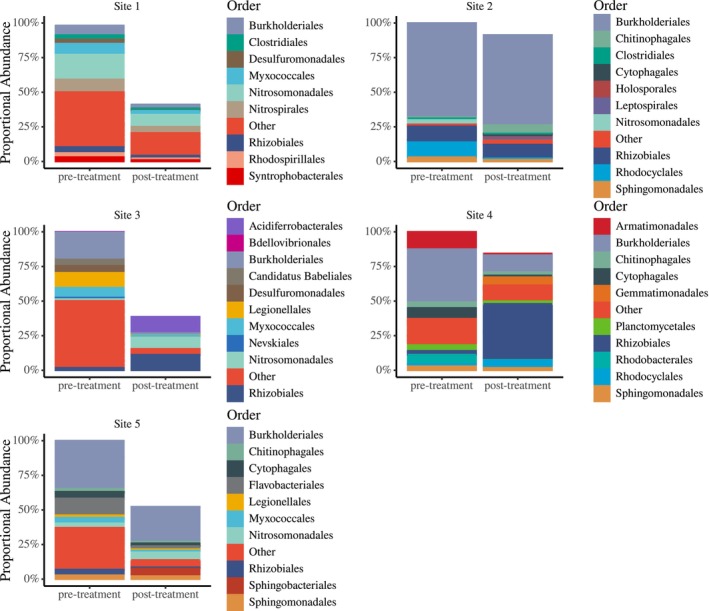
Proportional abundance of bacterial communities in pre‐ and post‐treatment water samples. *Note:* The top 10 most abundant orders are displayed, and the rest are denoted as “Other”. Water source types by Site; 1, 2, and 3 use groundwater and 4 and 5 use surface water.

There were five sites where pre‐ and post‐treatment water were collected from the same site. Figure 3 shows that the proportional abundance of bacterial identifications in post‐treatment water samples relative to pre‐treatment samples underwent a substantial reduction for supplies at Site 1, 3, and 5, and a minor reduction at Site 4. The reduction in the number of bacterial species detected in these supplies suggested that the treatment systems and/or source water protections were functioning appropriately to lower the bacterial hazard to human health. This was supported by non‐detection of *E. coli* and total coliforms by culturable methods in the post‐treatment drinking water. Conversely, the abundance of bacterial identifications increased for Site 2.

Neither *E. coli* nor total coliforms were detected in the pre‐ or post‐treatment samples taken from Site 1 (groundwater). The bacterial community structure was similar in both samples, but the post‐treatment sample had a reduction in the number of bacterial species identified (*n* = 48 bacterial species) when compared with the pre‐treatment sample (*n* = 162).

Both *E. coli* and total coliforms were detected in the pre‐treatment sample for Site 3 (groundwater) but were effectively inactivated during treatment. The bacterial species identified in the source water (*n* = 285) were mostly consistent with a freshwater environment, although a low concentration of bacteria associated with fecal environments and a low concentration of the fecal source ruminant marker was identified (Table S1). Fewer bacterial species were identified in the post‐treatment sample (*n* = 122) and no *E. coli* , total coliforms, or other bacteria harmful to human health were found.

The pre‐treatment supply at Site 5 (surface water) also contained *E. coli* and total coliforms, with relatively more bacterial species than other samples (*n* = 316). Over half of the bacteria identified were present at less than 1% abundance. The post‐treatment sample was generally consistent with a freshwater environment; however, several of the bacteria identified were indicative of fecal pollution. The general fecal source marker was detected but the concentration was below the limit of quantification and there was no detection of a specific animal fecal source. Neither *E. coli* nor total coliforms were detected in the post‐treatment sample, and none of the bacterial species identified (*n* = 197) were considered harmful.

No *E. coli* or total coliforms were detected in the pre‐ or post‐treatment sample from Site 2 (groundwater). A low number of bacterial species was identified in the pre‐treatment (*n* = 49) and post‐treatment (*n* = 54) samples. None of the bacteria were recognized as being harmful to human health and the types of bacteria identified in the community were consistent with a freshwater environment.

The pre‐treatment sample from Site 4 (surface water) contained both *E. coli* and total coliforms among the bacterial species (*n* = 85). The general fecal source marker was identified but specific animal or bird markers were absent. Some of the bacteria identified using metagenomics were also recognized as being harmful to human health, but these bacteria were not identified in the post‐treatment sample. The numbers of bacterial species decreased (*n* = 70) in the post‐treatment sample and neither *E. coli* nor total coliforms were identified. The limited reduction in proportional abundance between the pre‐ and post‐treatment samples and the types of bacteria indicated that a high biofilm bacterial community was present in the pipes.

### 3.4 Case Study: Action Taken to Resolve Contamination

**FIGURE 4:**
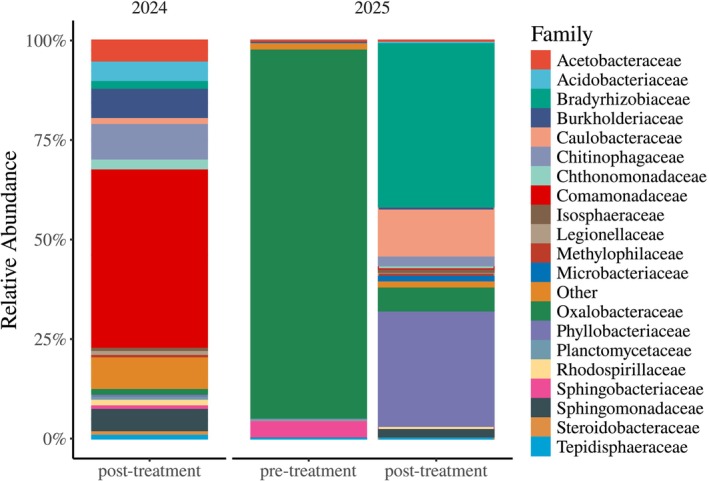
Relative abundance of matched pre‐ and post‐treatment samples before and after remedial intervention. *Note:* The top 10 most abundant orders are displayed with all others denoted as “Other”. No pre‐treatment sample was taken in 2024.

One private rainwater supply with UV treatment had repeated low‐level detections of *E. coli* (7.5–9.8 MPN/100 mL) and total coliforms (27–43 MPN/100 mL) as well as evidence of low‐level fecal contamination and *Legionella pneumophila* in the post‐treatment sample (Figure 4). Multiple samples were collected pre‐ and post‐treatment from various locations to identify the issue, including before and after UV treatment. The onsite observations and test results indicated that there was an intermittent issue with the treatment system. Further investigative testing revealed that the UV treatment efficacy declined with increasing water volumes, despite being within the specifications set out by the manufacturer.

Deep cleaning of the rooftop gutters and storage tank was carried out to reduce the buildup of sediment and other organic matter causing turbidity issues at lower water levels. In addition, a new UV treatment system was installed to overcome the intermittent issue. Subsequent water testing, including metagenomic analysis, identified no detections of *E. coli*, total coliforms, or evidence for *L. pneumophila* in post‐treatment samples. The new bacterial community was reflective of an early stage recolonization of taxa associated with plant and/or soil environments, indicative of the new filter media. Although the sampling indicated that treatment systems were functioning as expected after remedial interventions, further assessments could be required as treatment continues.

## 4 Discussion

### 4.1 Capability and Capacity Improvements

The Indigenous‐led, NTMP facilitated the training of 27 MWM community members and identified six supplies with microbial concerns. This demonstrates an effective approach to developing Indigenous operational capacity and identifying water quality hazards, which are required to better operate drinking water systems. Based on anecdotal feedback from the MWM, the program was pivotal in building soft infrastructure (knowledge and skills) within Indigenous communities. Collaboration and shared decision‐making within each Indigenous community ensured that the needs and priorities around capacity development were determined locally for the various supplies. For instance, the number of individuals engaged was greater than the number of communities in the program because there were multiple requests to enrol several members for online training and sampling.

The development of these skills often becomes people‐centered in community‐based programs (Henwood et al. 2019; Parlee et al. 2021). The collaborative approach helped to mitigate extractive research practices and developed foundational skills beyond data collection, necessary to facilitate longevity and continuity of Indigenous autonomy over water quality (Mercer et al. 2025). In addition, this program overcame common collection and transportation challenges for microbial sampling, which has been identified as the biggest barrier to monitoring remote sites (Rogers and Lawson 2024). The ability to regularly sample drinking water, due to newly established streamlined processes and local capability improvements, is now a compelling trait for further research with Ngāi Tahu community partners. This is exemplified by the external research funds that were granted to continue the program for an additional 4 years. Adopting an Indigenous‐led, collaborative governance approach ultimately improved the ability for these communities to access highly practical solutions and lasting drinking water improvements (Acharibasam et al. 2024).

### 4.2 Improved Drinking Water Autonomy

The NTMP facilitated an opportunity to better exercise rangatiratanga over drinking water supplies by leveraging new tools to understand drinking water hazards, which is key to advocating for responsible source water protection to regulatory authorities. While we did not empirically measure improvements in soft infrastructure, the anecdotal feedback from MWM was consistent with previous literature on Indigenous monitoring programs that showed enhanced public awareness, interest, and participation, as well as improved preparedness for drinking water safety and security (Buckland‐Nicks et al. 2016; Henwood et al. 2019). The program successfully engaged 18 Ngāi Tahu communities for water sampling and trained 27 local individuals from 16 of these. Furthermore, the NTMP proved essential in establishing baseline information and identifying hotspots where remedial interventions were required to strengthen Indigenous sovereignty and self‐determination, often where water supplies were interlinked with cultural practice (Acharibasam et al. 2024; Buckland‐Nicks et al. 2016). The full subscription of two recent government funds dedicated to improving rural and marae drinking water infrastructure in NZ (Taumata Arowai 2024) highlights the need and strong community demand to progress these safe water solutions. The combination of improvements to soft infrastructure and a persisting financial challenge underscores the benefit of establishing a systematic and streamlined water quality monitoring framework.

Persisting systemic and structural barriers, including remoteness (Nguyen et al. 2026), continue to constrain the assertion of Ngāi Tahu rangatiratanga and access to safe and secure drinking water (Henwood et al. 2019). Existing literature has suggested that the ongoing investment of resources, opportunities for training and partnership, and continued water quality testing are required to sustain the delivery of safe and secure drinking water (Balasooriya et al. 2023; Mercer et al. 2025). Additionally, partners and researchers have an important role to play in enhancing the capacity for Indigenous leadership to be integrated, which is particularly important when trialing new technologies and ensuring they are fit for purpose (Balasooriya et al. 2023; Wilson et al. 2024). The findings here contribute to a growing body of evidence that has demonstrated how greater Indigenous participation in decision‐making and water quality improvements can be reasonably achieved when Indigenous‐led programs are sufficiently resourced and supported (Acharibasam et al. 2024; Buckland‐Nicks et al. 2016; Henwood et al. 2019; Parlee et al. 2021; Wilson et al. 2018; Wilson et al. 2024).

### 4.3 Public Health Insights

The adequacy of hard infrastructure (treatment and storage procedures) was determined through routine water sampling and advanced sampling techniques. The routine drinking water quality results provided an overview of potential microbial and chemical hazards, while the use of novel tools, including qPCR and metagenomics for detecting fecal contamination in drinking water, provided complementary information about potential health hazards.

#### 4.3.1 Public Water Supplies

In general, the sites reliant on public drinking water supplies that did not have any on‐site water storage were compliant with current drinking water standards. Despite these sites being compliant with drinking water standards, some of the routine testing results were misaligned with Indigenous community aspirations. For example, community concerns have been expressed about the nitrate levels in one supply that sporadically breached the drinking water standard in a previous year (Prickett et al. 2024). This occurred at a concentration above those observed in emerging epidemiological evidence that links elevated nitrate intake to several adverse health outcomes (Royal et al. 2024).

#### 4.3.2 Private and Public‐Private Water Supplies

In contrast to the sites relying on public drinking water supplies, there were several microbial hazards identified in the public‐private and private drinking water supplies. At the sites storing water on site from a public supply, there was a disconnect concerning who had the responsibility for managing water quality and the potential hazards associated with localized storage. Additionally, there were private supplies that did not have adequate treatment in place, which subsequently increased vulnerability to microbial hazards.

In 2024, more than half of the laboratory notifications of *E. coli* detections reported to Taumata Arowai|The Water Services Authority were from self‐supplied schools, which are often rural‐ or remote‐based supplies (Taumata Arowai 2024). Marae experience similar challenges to rural schools, including the need to host a fluctuating number of people. These sentinel sites are vulnerable to similar source water hazards and treatment capacity challenges. Rogers and Lawson (2024) have highlighted that *E. coli* was the most at‐risk determinand of environmental concern for drinking water in rural NZ. Specifically, they found bacterial exceedances occurred in many rural schools, regardless of water‐treatment systems, indicating that the operation or maintenance of these systems was ineffective.

The microbial exceedances presented here mirror the challenges faced by rural schools and indicate that there are likely to be other marae and papakāinga across NZ that face similar enteric disease hazards. This is illustrated by the remedial intervention that required additional maintenance to be carried out and the installation of replacement parts, despite meeting manufacturer's guidelines, to ensure the safe operation of a water system. Furthermore, our findings are consistent with Henwood et al. (2019) who found some remote North Island Māori communities were exposed to significant *E. coli* exceedances from private roof and tank water supplies. This water safety challenge is evidently borne by remote self‐supplied communities, particularly those that have limited ability to influence local land use/source water pollution or do not have the financial, or operational capacity to act on contaminant hazards. This improved understanding of water‐related hazards could be used to better inform the prioritization of remote drinking water infrastructure investments to ensure safe drinking water is achieved for all (Nguyen et al. 2026).

#### 4.3.3 Advanced Techniques

Indigenous monitoring programs that aim to incorporate novel techniques are stronger when practiced in partnership or with the support of specialist entities. For instance, detecting low levels of the pathogenic bacterium *L. pneumophila* using metagenomics enabled an Indigenous community to make informed decisions about the management of a water supply and reduce health hazards. Metagenomics is a promising tool for drinking water surveillance when used in association with regulatory methods for detecting viable fecal indicator bacteria. However, the need for highly specialized laboratory skills and equipment for metagenomics poses a technical barrier for many Indigenous communities. As such, a careful approach is required during the generation and dissemination of empirical findings to ensure meaningful outcomes.

Metagenomics remains in the developmental stage for this application and there are ongoing questions about what a “positive” result means. A challenge when using metagenomic techniques targeting bacterial DNA is that detection of the bacterial species does not differentiate between live and dead cells (Guo et al. 2021; Kahlisch et al. 2012; Lindner et al. 2024). This is important when investigating post‐treatment drinking water as detected sequences may identify nonviable bacterial species inactivated by treatment processes. In this study, the viability aspect and health hazard are addressed by using methods that identify viable fecal indicator bacteria (total coliforms and *E. coli* ) in the treated drinking water, concurrent with qPCR and metagenomic techniques to provide multiple lines of evidence for interpretation.

In addition, we are refining methods to improve the identification of bacteria that may not be resolved at the species level by the full‐length 16S rRNA gene used herein. References are being developed to overcome the poor representation in current databases, which are weighted toward bacteria relevant to human health rather than closely related environmental bacteria. Additional tools for confirming taxonomic identities at a greater resolution include full metagenomic (shotgun) assays and qPCR targets for specific bacteria of interest, including *L. pneumophila* .

### 4.4 Collaborative Partnerships

Intercultural collaborations are stronger and lead to enhanced rigor, relevance, and accessibility when trusting relationships have been formed (Mercer et al. 2025; Wilson et al. 2018). Parlee et al. (2021) have highlighted the importance of ensuring Indigenous communities have data sovereignty over the intellectual property produced from monitoring their water supplies. Data sovereignty must also entail authoritative powers because the way data are interpreted and integrated is dependent on worldviews and will inform subsequent decision‐making processes (Mercer et al. 2025; Wilson 2025). Addressing these constraints intentionally provides a unique opportunity for both parties to exchange different forms of knowledge and to produce a more holistic understanding of water quality.

The Indigenous Observation Network established by the Yukon River Inter‐tribal Watershed Council found their community‐based water quality monitoring network valuable for data credibility and building trust to mobilize data in decision‐making processes. Wilson et al. (2018) described the alignment with community values, independence of colonial government mandate, and sense of ownership over data as strengths of this approach. Often it is the colonial frameworks themselves that create the structural conditions that demand Indigenous‐led monitoring programs to be established (Wilson 2025). To address this challenge, additional governmental support is required, where appropriate and feasible, for Indigenous communities to understand drinking water hazards, upskill people locally, and access infrastructure upgrades. In the NZ context, support for Māori drinking water supplies is consistent with the Government's obligations under the Treaty of Waitangi, the country's founding document intended to provide Māori with equitable service provision and health outcomes, among many other guarantees. The NTMP subsequently offers a decolonial opportunity to water governance where existing frameworks have failed to adequately recognize and respect Indigenous perspectives and values (Parlee et al. 2021).

### 4.5 Strengths and Limitations

The NTMP was Indigenous‐led and co‐designed with Indigenous communities and organizations, academic institutions, and water sector entities. The NTMP, resultantly, had a high uptake from the majority of Ngāi Tahu communities across their takiwā that allowed for a comprehensive understanding of water quality and the appropriate resolution of issues when they were identified. Additionally, the NTMP produced a large cohort of skilled MWM who continue to be pillars of freshwater knowledge within their local communities.

A key limitation of this study was the cost of establishing and maintaining the NTMP that may limit its generalizability to other jurisdictions. It was developed and maintained primarily through funding from competitive research grants, although the material, method of engagement, and sampling procedure that have been developed through this project can now be applied to other regions through new monitoring programs. Another limitation of this study is the small number of sampling periods, particularly for the qPCR and metagenomic testing. For example, metagenomic data were collected from one point in time and could not be leveraged to full potential. The metagenomic section of this study provided proof‐of‐concept for a larger study that is currently underway and will monitor these same sites over a three‐year period with a quarterly interval of sampling each year. This expanded dataset will investigate the potential of metagenomic analysis for detecting changes in microbial communities over time and its validity for indicating issues with treatment efficacy or microbial contamination that would not be detected via standard fecal indicator testing (Phiri et al. 2021; Taylor et al. 2024).

## 5 Conclusions

An Indigenous‐led drinking water quality program supported the recruitment and training of local community members. The NTMP underscores the value of an Indigenous‐led approach to drinking water management and the benefit of removing systemic barriers that cause disparities in access to quality water services. The program has led to tangible improvements in drinking water quality, developed operational capabilities within Indigenous communities, and reduced inequitable human health hazards. The use of a more sophisticated drinking water laboratory test for microbial contamination shows promise for a better understanding of source water hazards and treatment efficacy for drinking water supplies.

## Author Contributions

**Connor Redmile:** conceptualization, data curation, funding acquisition, investigation, methodology, writing – original draft, writing – review and editing. **Donna Sutherland:** data curation, investigation, methodology, writing – review and editing. **Megan Devane:** conceptualization, data curation, formal analysis, funding acquisition, methodology, visualization, writing – review and editing. **William Taylor:** data curation, formal analysis, funding acquisition, methodology, visualization, writing – review and editing. **Isobel Busby:** project administration, visualization, writing – original draft, writing – review and editing. **Anne Glackin:** conceptualization, funding acquisition, investigation, methodology, project administration, writing – review and editing. **Brent Gilpin:** conceptualization, formal analysis, funding acquisition, methodology, writing – review and editing. **Tim Chambers:** conceptualization, data curation, formal analysis, funding acquisition, project administration, methodology, visualization, writing – original draft, writing – review and editing.

## Funding

This work was supported by the Ministry of Business, Innovation and Employment ESR2411 and TN/PWC/19/UoOWTC.

## Conflicts of Interest

The authors declare no conflicts of interest.

## Supporting information


**Table S1:** Microbial source tracking markers detected in drinking water samples.

## Data Availability

Research data are not shared.
