# Supplementary material for: The Establishment of an Indigenous‐Led Drinking Water Monitoring Program Leveraging qPCR and Metagenomics Testing in New Zealand
Source: Water Environ Res. 2026 Jun 27;98(7):e70471. doi: 10.1002/wer.70471 (PMC13309855; doi:10.1002/wer.70471)
Supplement: Supplementary file 1 — Table S1: Microbial source tracking markers detected in drinking water samples. [file WER-98-e70471-s001.docx]

Contents

[Introduction 2](#_Toc229469443)

[Sampling Protocol 2](#_Toc229469444)

[Microbial Source Tracking Markers Detected in Drinking Water Samples 8](#_Toc229469445)

# Introduction

The following document outlines the sampling protocol and the microbial source tracking markers detected in the pre- and post-treatment samples. The protocol is a consolidation of established approaches from local water testing laboratories, including Christchurch City Council (New Zealand) and Hill Labs. The protocol was designed for programme participants to follow to ensure greater rigor and consistency, despite their varying backgrounds and levels of experience.

The microbial source tracking markers/results are displayed for total coliforms, *Escherichia coli,* general, human, ruminant, and avian indicators. Table S1 presents the results from sampling, including the volume of water that was filtered and the number of gene copies detected.

# Sampling Protocol

The following pages outline the sampling protocol that was developed for Water Champions/Māngai Wai Māori to follow when conducting water quality testing.

**Water Quality Testing Protocol:**

PHASE ONE: PREPARATION

Please ensure that the sample, once collected, can be delivered to Hill Labs within 24 hours, this is particularly important if courier services are required.

Before completing a routine drinking water sample, the **PREPARATION** section should be completed. This is essential to ensure the testing process is accurate, repeatable, and well recorded.

Find a suitable drinking water source. This should be easily accessible and can be a commonly used tap. Make sure your hands are clean and that gloves are worn when testing. This protects the sample and sampler from exposure to waterborne pathogens or chemical residue.

If there are treatment devices connected to the supply at a later point (i.e., an under-sink filter) then ensure the water after this extra treatment is used in the post-treatment sample. If you are sampling the source water, please also complete a pre-treatment test.

**PLEASE COMPLETE THE BELOW SECTION**

Sampler Name(s):

Sample Name (i.e., Moeraki Kitchen) ……………………………………. | (Post-treatment)

Sample No. …One… Date ……..../.....…../………. Start time ………. am / pm

*If completing a Pre-treatment sample, also record the following:*

Sample Name (i.e., Moeraki Outside) ……………………………………. | (Pre-treatment)

Sample No. …Two… Date ……..../.....…../………. Start time ………. am / pm

**Supply Source** (circle): Groundwater Rainwater Surface water

Comments (this can include any detail on recent supply maintenance i.e., storage tank or roofing was cleaned, recent weather i.e., heavy rainfall or dry and sunny, complaints about water quality i.e., taste, colour, odour):

……………………………………………………………………………………………………………………………………………….………………………………………………………………………………………………………………………………………………..…………………

**CHECKLIST**:

- Disposable nitrile gloves (powder free) and clear safety glasses
- Alcohol wipes (for sterilisation)
- Chilly box with frozen cooler pads (store ice packs in freezer until sampling)
- 400 mL sterile container (containing sodium thiosulfate)
- 500 mL unpreserved container
- 100 mL nitric acid preserved container (Caution: nitric acid is very corrosive)
- Hill Labs Analysis Request Form / Chain of Custody Form (pre-filled)
- Supplied address label and pre-paid courier ticket

PHASE TWO: SAMPLING

The second phase of this process should be strictly followed. There is a high chance that the sample can become contaminated, so it is important that this risk is reduced. First read through Phase Two and Three before continuing and watch the sampling video.

**Pre-sample Check**

Ensure you have completed Phase One and have everything under **CHECKLIST**.

Organise the delivery aspects of the sample before you begin. If you require same day delivery, sample early in the morning; if you require overnight delivery, sample early in the afternoon. Plan to sample on Monday, Tuesday, or Wednesday.

**Tap Flushing**

Remove any fittings from the tap (i.e., hose fittings) as they may hold bacteria.

The tap should be turned on to a moderate flow to flush water for at least 2 minutes. This will help to remove the build-up of any heavy metals that can accumulate in the tap. Feel free to save this water for external purposes.

*Ensure that your hands are clean, and the tap is off before Sterilisation.*

**Sterilisation**

The area around the tap should be clear of anything that can contaminate or be contaminated by the tap (i.e., food, rubbish, animals).

Using the alcohol wipe, thoroughly clean the tap to ensure any bugs are killed. Complete this for 30 seconds to allow the chemicals enough time to interact.

After you have finished sterilising, turn the tap back on to a flow that will allow you to fill the container without splashing or overfilling. This will be a relatively slow flow and will flush out any remaining chemical residue. Try not to adjust this flow again.

**Sampling**

Disposable examination gloves and eye protection (glasses or sunglasses are okay) should be worn when handling the samples and chemicals. Please take extra care when putting gloves on to prevent contamination with your bare hands.

On the closed sample container, record the sample name, date, time of sampling, and any other details it requests.


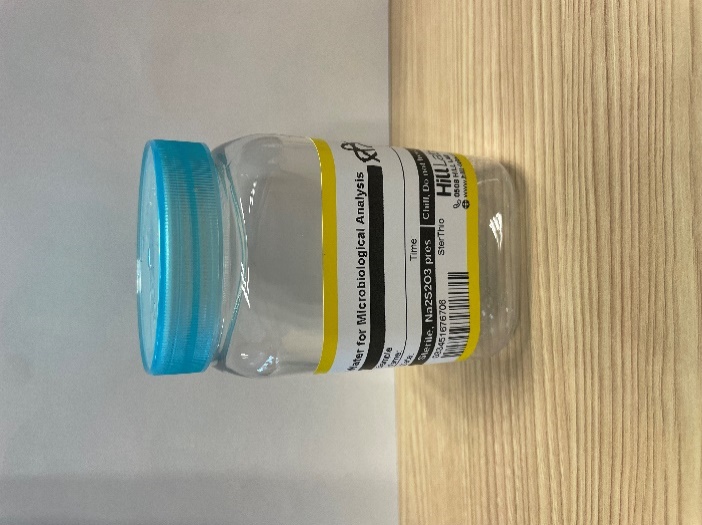

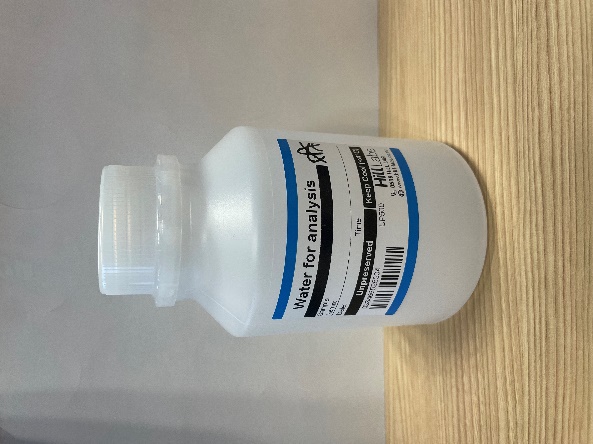

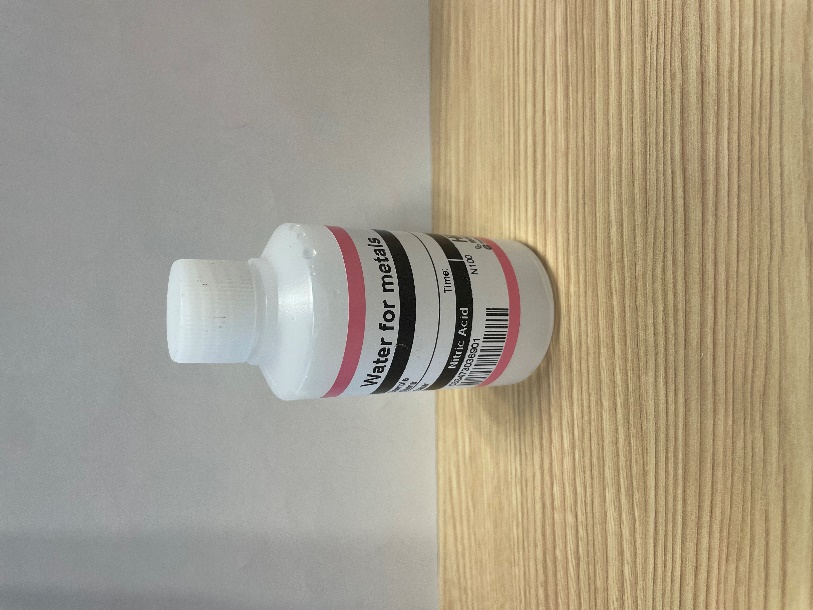


500 mL Routine water sample container

100 mL Nitric acid preserved container

400 mL Microbiological sample container

If you are taking a microbiological sample, then do not rinse the container. There will be a chemical residue inside to neutralise the effect of chlorine. Ensure the bottle remains closed for as long as possible — the surrounding air can carry pathogens.

Hold the 400 mL sample container near the base and bring it to the tap. Unscrew the lid and keep the lid facing down. Immediately begin to fill the container. Try to keep the lid close and do not let the container or lid touch the tap nozzle.

Fill the container up being sure to leave around 1–2 cm of airspace inside. Tightly screw the lid back on. Immediately transfer the sample to the chilly box.

The chilled cooler pads should already be in the chilly box, and the sample should be separated from these by the layer of bubble wrap to avoid direct contact. The sample should remain chilled (but not frozen) and in the dark during its transit to the lab.

Take the routine water sample next. Avoid rinsing this container.

Hold the 500 mL sample container near the base and bring it to the tap. Unscrew the lid and begin filling the container. Fill this container entirely and do not leave an air gap. Tightly screw the lid on and place the sample in the chilly box. Ensure both samples are separated from the cooler pads with bubble wrap.

Take the 100 mL nitric acid preserved container. Be sure to take care as nitric acid is very corrosive and dangerous. Do not rinse the container.

Hold the container near the base and bring it to the tap. Unscrew the lid and begin filling the container. Fill the container up being sure to leave around 1-2 cm of airspace inside. Do not spill the sample contents. Tightly screw on the lid. Transfer the sample to the chilly box.

Carefully remove your gloves making sure to avoid touching areas that have been in contact with water. Wash your hands thoroughly.

Turn the tap off. Make sure that you have labelled all the sampling bottles with the appropriate details. These details ensure the correct identification. Fill out the Hill Labs Analysis Request form and place it inside the supplied envelope, leaving it inside the chilly box on top of the chilled samples.


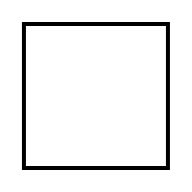


I have correctly recorded the details of the sampling bottles (End time ………. am / pm)

PHASE THREE: DELIVERY

The third phase of this process includes sample delivery and analysis. It is important that a sample is delivered to a lab within 24 hours otherwise the results are unreliable.

Secure the lid of the chilly box tightly with tape. Attach the supplied address label and pre-paid courier ticket to the top of the chilly box.

Deliver the sample to the lab as soon as possible. The sample must arrive at the lab within 24 hours, within 6 hours is preferable, and the sample must not exceed 10^o^C — so ensure the ice packs are properly frozen before sampling.

If you are located nearby, drop the chilly box off directly to Hill Labs. If you live further away, drop the chilly box off to the nearest NZCourier Agent or Depot using the list provided.

Once the samples reach the lab, the lab technicians will process the samples and provide the results using the information you have supplied. It may take several working days for the results to come through.

Once you have finished this process, send us a copy of this completed Protocol as confirmation that the testing has been completed. This will help ensure the correct identification of samples for each location.


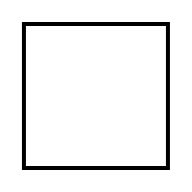
I have provided the samples to the lab /courier / the courier has collected the samples

(Drop-off / Collection time ………. am / pm)

**HEALTH AND SAFETY**

If a Health and Safety incident occurs, you must report this. This includes any near misses or small incidents, fears or concerns around sampling, as well as any serious incidents that occur.

**Chemical and Pathogenic Risk**

It is essential the person handling samples wears appropriate personal protective equipment. This reduces risk of injury. Please wear appropriate gloves and glasses when sampling.

The handler should ensure flow of water does not cause nitric acid or sodium thiosulfate to splash out of container and that containers are handled responsibly to prevent chemical spills.

Nitric acid is a preservative for metal in water and is corrosive. Can cause severe burns, eye damage and respiratory irritation. See the provided Safety Data Sheet for nitric acid for detailed hazard analysis.

If nitric acid gets into eye(s), immediately flush with large amounts of water for at least 30 minutes, lifting upper and lower eyelids. Remove contact lenses if worn. Seek medical attention.

If nitric acid gets on skin, quickly remove contaminated clothing, and immediately wash contaminated skin with large amounts of water. Seek medical attention.

If nitric acid is inhaled, remove person from exposure. Begin rescue breathing if breathing has stopped and CPR if heart action has stopped. Transfer promptly to a medical facility.

Sodium thiosulfate is used to dechlorinate tap water and ensures that microorganism samples are representative of the time they were sampled. See the provided Safety Data Sheet for sodium thiosulfate for detailed hazard analysis.

If sodium thiosulfate is inhaled, move exposed individual to fresh air. Loosen clothing as necessary and position individual in comfortable position. Seek medical advice if discomfort or irritation persists. If breathing is difficult, give oxygen.

If sodium thiosulfate gets on skin, wash affected area with soap and water. Seek medical advice is discomfort or irritation persists. Flush skin with plenty of soap and water for at least 15 minutes.

While the water being sampled is for consumptive purposes, there is a risk of pathogenic contamination that can lead to sickness. This may occur when sampling water prior to treatment. Do not consume untreated water, practice good hygiene, and thoroughly wash hands after handling any water before eating or drinking.

#

# Microbial Source Tracking Markers Detected in Drinking Water Samples

The below table presents the microbial source tracking markers detected in pre- and post-treatment drinking water samples, expressed as the number of gene copies per 100 mL for each site sampled.

Supplementary Table S1.

| Test ID | Site No. | Date | Time | Treatment status | Volume filtered (mL) | Total coliforms  (MPN /  100 mL) | *E. coli*  (MPN /  100 mL) | General GenBac | Human HF183 | Human crAssPhage | Human BiADO | Ruminant BacR | Avian GFD |
| --- | --- | --- | --- | --- | --- | --- | --- | --- | --- | --- | --- | --- | --- |
| 1 | 1 | 22/10/2024 | 10:50 | pre | 2000 | <1.0 | <1.0 | <21 | <15 | <50 | <21 | <18 | <14 |
| 2 | 1 | 22/10/2024 | 10:40 | post | 2000 | <1.0 | <1.0 | <21 | <15 | <50 | <21 | <18 | <14 |
| 3 | 2 | 4/11/2024 | 9:15 | pre | 2000 | <1.0 | <1.0 | <21 | <15 | <50 | <21 | <18 | <14 |
| 4 | 2 | 4/11/2024 | 9:00 | post | 2000 | <1.0 | <1.0 | <21 | <15 | <50 | <21 | <18 | <14 |
| 5 | 3 | 12/11/2024 | 10:00 | pre | 1000 | 43 | 12 | 940 | <15 | <50 | <21 | 38 | <14 |
| 6 | 3 | 12/11/2024 | 8:15 | post | 1000 | <1.0 | <1.0 | <21 | <15 | <50 | <21 | <18 | <14 |
| 7 | 4 | 5/11/2024 | 12:20 | pre | 2000 | 190 | 9.8 | 1000 | <15 | <50 | <21 | <18 | <14 |
| 8 | 4 | 5/11/2024 | 12:00 | post | 2000 | <1.0 | <1.0 | <21 | <15 | <50 | <21 | <18 | <14 |
| 9 | 5 | 21/10/2024 | 11:30 | pre | 2000 | 550 | 27 | 2,900 | <15 | <50 | <21 | <18 | <14 |
| 10 | 5 | 21/10/2024 | 11:30 | post | 2000 | <1.0 | <1.0 | DNQ | <15 | <50 | <21 | <18 | <14 |
| 11 | 6 | 21/10/2024 | 13:20 | post | 2000 | <1.0 | <1.0 | <21 | <15 | <50 | <21 | <18 | <14 |
| 12 | 6 | 21/10/2024 | 14:00 | post | 2000 | 16 | <1.0 | 5300 | <15 | <50 | <21 | 22 | <14 |
| 13 | 7 | 5/11/2024 | 13:00 | post | 2000 | <1.0 | <1.0 | <21 | <15 | <50 | <21 | <18 | <14 |
| 14 | 7 | 5/11/2024 | 13:30 | post | 2000 | 3.1 | <1.0 | <21 | <15 | <50 | <21 | <18 | <14 |
| 15 | 8 | 4/11/2024 | 9:48 | post | 2000 | <1.0 | <1.0 | <21 | <15 | <50 | <21 | <18 | <14 |
| 16 | 9 | 2/12/2024 | 14:50 | post | 2000 | <1.0 | <1.0 | <21 | <15 | <50 | <21 | <18 | <14 |
| 17 | 10 | 5/11/2024 | 7:35 | post | 2000 | <1.0 | <1.0 | 190 | <15 | <50 | <21 | <18 | <14 |
| 18 | 10 | 5/11/2024 | 8:00 | post | 1500 | <1.0 | <1.0 | <21 | <15 | <50 | <21 | <18 | <14 |
| 19 | 11 | 25/11/2024 | 10:00 | post | 2000 | 27 | 7.5 | 130 | <15 | <50 | <21 | <18 | <14 |
| 20 | 11 | 25/11/2024 | 10:00 | post | 2000 | 43 | 9.8 | 90 | <15 | <50 | <21 | <18 | <14 |
| 21 | 11 | 13/08/2025 | 11:30 | post | 2000 | <1.0 | <1.0 | 430 | <15 | <50 | <21 | <18 | <14 |
| 22 | 11 | 13/08/2025 | 11:30 | pre | 2000 | <1.0 | <1.0 | 1400 | 32 | <50 | <21 | <18 | <14 |
| 23 | 12 | 12/11/2024 | NA | post | 1000 | <1.0 | <1.0 | <21 | <15 | <50 | <21 | <18 | <14 |
| 24 | 13 | 12/12/2024 | 15:50 | post | 2000 | <1.0 | <1.0 | <21 | <15 | <50 | <21 | <18 | <14 |
| 25 | 14 | 4/11/2024 | 11:31 | no treatment | 2000 | 770 | 18 | <21 | <15 | <50 | <21 | <18 | <14 |
| 26 | 14 | 4/11/2024 | 11:36 | no treatment | 2000 | 980 | 16 | <21 | <15 | <50 | <21 | <18 | <14 |
| 27 | 15 | 17/12/2024 | 12:00 | post | 2000 | <1.0 | <1.0 | <21 | <15 | <50 | <21 | <18 | <14 |
| 28 | 15 | 17/12/2024 | 12:15 | post | 1700 | not tested | not tested | <21 | <15 | <50 | <21 | <18 | <14 |
| 29 | 16 | 5/11/2024 | 11:13 | post | 2000 | <1.0 | <1.0 | <21 | <15 | <50 | <21 | <18 | <14 |
| 30 | 17 | 4/11/2024 | 12:14 | post | 2000 | 24 | <1.0 | 67 | <15 | <50 | <21 | DNQ | <14 |
| 31 | 17 | 13/11/2024 | 7:46 | post | 1000 | 6.3 | 1.0 | 38 | <15 | <50 | <21 | <18 | <14 |
| 32 | 18 | 21/10/2024 | 08:35 | post | 2000 | <1.0 | <1.0 | <21 | <15 | <50 | <21 | <18 | <14 |

*Note*. All microbial source tracking markers are reported as gene copies per 100 mL of water. MPN, Most probable number. The “<” symbol denotes the limit of quantification in the volume of water filtered. DNQ, detected not quantified; marker detected in all replicates but below the limit of quantification.
